# Anti-rhesus D prophylaxis in pregnant women is based on sialylated IgG antibodies

**DOI:** 10.12688/f1000research.2-169.v1

**Published:** 2013-08-09

**Authors:** André Winkler, Markus Berger, Marc Ehlers

**Affiliations:** 1 Laboratory of Tolerance and Autoimmunity, German Rheumatism Research Center (DRFZ), Leibniz Institute, Berlin, 10117, Germany; 2 Laboratory of Tolerance and Autoimmunity, Institute for Systemic Inflammation Research, University of Luebeck, Luebeck, 23538, Germany; 3 Laboratory of Glycodesign and Glycoanalytics, Institute for Laboratory Medicine, Clinical Chemistry and Pathobiochemistry, Charité – University Medicine Berlin, Berlin, 10117, Germany

## Abstract

Red blood cells (RBCs) from a rhesus D (RhD)-positive fetus that reach the bloodstream of an RhD-negative pregnant woman during birth can induce a pathogenic antibody (Ab) response against the RhD-positive RBCs, leading to fetal hemolytic disease in subsequent pregnancies. To prevent a pathogenic immune reaction, the RhD-negative mother receives serum immunoglobulin G (IgG) containing polyclonal RhD-specific IgG Abs that is purified from healthy RhD-negative men immunized with RhD-positive RBCs. However, the protective mechanism of these polyclonal RhD-specific IgG Abs is unclear. It has become increasingly clear that the effector function of IgG Abs is regulated by the glycan pattern linked to the Fc region of IgG Abs. Non-fucosylated (afucosylated) IgG Abs have a higher affinity for activating Fc gamma receptors, and thus induce a stronger Ab-dependent cellular cytotoxicity (ADCC) reaction than do fucosylated IgG Abs. Agalactosylated and asialylated, autoantigen-specific serum IgG Abs correlate with pro-inflammatory immune responses and disease activity in patients with rheumatoid arthritis. In contrast, galactosylated and sialylated IgG Abs are immunosuppressive and inhibit in form of immune complexes (ICs) dendritic cell (DC) maturation and pro-inflammatory T and B cell immune responses in an antigen-specific manner. However, the galactosylation and sialylation levels of the protective polyclonal RhD-specific IgG Abs are unknown. Here, we purified RhD-specific IgG Abs from the approved commercial product Rhophylac® (CSL Behring) and found that these RhD-specific IgG Abs were even more galactosylated and sialylated than the total Rhophylac® IgG Abs. This result suggests that these galactosylated and sialylated polyclonal RhD-specific IgG Abs are immunosuppressive and induce tolerance against RhD, which would be in strong contrast to a low fucosylated, low galactosylated and low sialylated monoclonal RhD-specific IgG Ab developed to prevent fetal hemolytic disease that has recently passed a clinical phase II study.

## Introduction

Red blood cells (RBCs) from a rhesus D (RhD)-positive fetus that get in contact with immune cells of an RhD-negative pregnant woman during birth can induce a pathogenic antibody (Ab) response against the RhD-positive RBCs, leading to fetal hemolytic disease in subsequent pregnancies with RhD-positive fetuses after transplacental passage. To prevent allo-immunization by RhD-positive fetal RBCs, the RhD-negative mother receives one prenatal and one postnatal injection of serum immunoglobulin G (IgG) containing polyclonal RhD-specific IgG Abs that is purified from healthy RhD-negative men immunized with RhD-positive RBCs. Such a passive anti-RhD IgG Ab treatment i) induces a rapid clearance of RhD-positive RBCs from the bloodstream of the mother and ii) inhibits the development of pathogenic anti-RhD Abs by the mother ^1^.

However, the protective mechanism of passive anti-RhD treatment remains unclear. It is hypothesized that the clearance of RhD-positive RBCs is mediated through an Fcγ receptor (Fcγ R) IIIA-mediated Ab-dependent cellular cytotoxicity (ADCC) reaction and that rapid clearance prevents immunization ^1– 4^. To prevent immunization, it has also been suggested that polyclonal anti-RhD IgG Abs have to inhibit the activation of RhD-specific B cells through the co-ligation of the B cell receptor and an inhibitory receptor, such as the IgG inhibitory receptor FcγRIIB ^2, 3^. Furthermore, tolerance induction via antigen-presenting cells (APCs) has been posited to inhibit pro-inflammatory, RhD-specific T cell responses ^2, 5, 6^. However, it is questionable whether FcγRIIIA crosslinking on APCs inhibits pro-inflammatory RhD-specific T cell responses but rather enforces pro-inflammatory T cell responses ^7^. It is more likely that polyclonal anti-RhD IgG Abs target an inhibitory receptor (complex) on APCs to induce regulatory T cells and tolerance for inhibiting pro-inflammatory T cell and therewith also T cell-depemdent B cell responses.

Attempts to substitute this polyclonal anti-RhD IgG prophylaxis with RhD-specific monoclonal IgG Abs have failed because the monoclonal RhD-specific IgG Abs were relatively unstable due to intramolecular rearrangements or did not clear RhD-positive RBCs as rapidly as the available polyclonal anti-RhD IgG Abs in *in vitro* assays or clinical trials and/or did not sufficiently inhibit allo-immunization in clinical trials ^1, 4, 8^.

**Figure 1.**
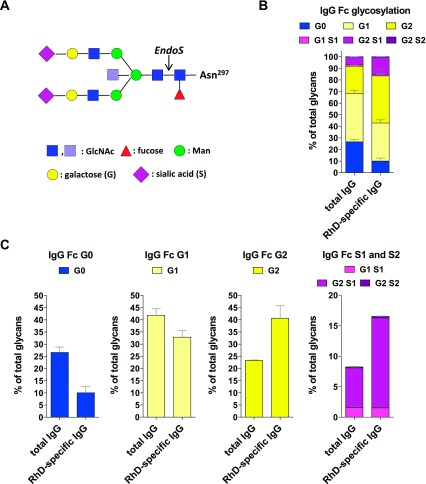
Polyclonal RhD-specific IgG Abs in Rhophylac® are sialylated. ( **A**) The biantennary IgG Fc glycan core structure, which is coupled to Asn 297, consists of two N-acetyl-glucosamines (GlcNAc; dark blue) and three mannoses (Man), which can be further decorated with fucose; bisecting GlcNAc (light blue) and terminal GlcNAc (dark blue), galactose (G) and sialic acid (S). ( **B** and **C**) The purified total and RhD-specific IgG samples were hydrolyzed with EndoS and analyzed by MALDI-TOF MS. The cleavage site of EndoS is indicated by an arrow in ( **A**). ( **B**) The bar graph indicate the frequency of all glycan structures with 0, 1 or 2 galactose (G) residues and 0, 1 or 2 sialic acid (S) residues. The mean values with the standard error of the mean (SEM) from independent experiments are shown. ( **C**) The bar graphs separately indicate the frequency of G0, G1, G2 and S1 and S2 glycan structures from the bar graph in ( **B**).

It has become increasingly clear that the effector function of IgG Abs is highly regulated by the Abs’ Fc N-linked glycosylation pattern ( Figure 1A). Non-fucosylated (afucosylated) IgG Abs have a higher affinity for activating FcγRs, such as FcγRIIIA, and thus induce a stronger ADCC reaction than do fucosylated IgG Abs ^9, 10^. This finding is currently translated, for example, into tumor-specific Ab therapy.

Agalactosylated and asialylated (G0), autoantigen-specific serum IgG Abs correlate with pro-inflammatory immune responses and disease activity in patients with rheumatoid arthritis (RA) ^11– 14^. In contrast, pregnancy-induced and anti-tumor necrosis factor (TNF) therapy-induced remission in RA patients is associated with an increase in galactosylated and sialylated IgG Abs ^15, 16^. In this context, an anti-inflammatory role has been suggested for sialylated IgG Abs. Accordingly, the sialylated IgG subfraction of intravenous IgG (IVIG), which is purified from pooled human plasma from healthy donors and used to systemically treat autoimmunity in high doses (2 g/kg), exhibits anti-inflammatory activity ^17– 20^. We have recently shown that low doses of immune complexes (ICs) containing sialylated antigen-specific IgG Abs inhibit dendritic cell maturation and pro-inflammatory T and B cell immune responses in an antigen-specific manner ^21– 23^. ICs containing 15%, but not 5%, sialylated IgG Abs have further been sufficient to inhibit B cell activation *in vitro* ^23^. Furthermore, it has recently been shown that ICs containing galactosylated, but not sialylated, IgG Abs are already sufficient to inhibit neutrophil activation ^24^.

Thus, G0 IgG Abs enhance, whereas galactosylated and sialylated IgG Abs suppress, pro-inflammatory immune responses. Accordingly, ICs containing galactosylated and sialylated IgG Abs inhibit rather than induce an ADCC reaction by at least reduced binding affinity of galactosylated and sialylated IgG Abs to FcγRIIIA ^17, 25^ but also likely by active suppression mechanisms via inhibitory receptors on immune cells.

However, based on the assumption that anti-RhD IgG Abs clear RhD-positive RBCs through an ADCC reaction, the different outcomes of monoclonal anti-RhD IgG Abs have been particularly attributed to different levels of fucose ^1, 2, 4, 26– 29^. By contrast, the role of anti-RhD IgG galactosylation and sialylation has hardly been investigated.

Based on the idea that anti-tumor as well as anti-RhD IgG Abs should induce a strong ADCC response by recruiting FcγRIIIA-expressing immune cells, the French biotechnology company Laboratoire Francais du Fractionnement et des Biotechnologies (LFB; Les Ulis, France) has generated a monoclonal anti-CD20 IgG1 Ab (ublituximab; LFB-R603) and a human monoclonal RhD-specific IgG1 Ab (roledumab, LFB-R593 ^30^) with low Fc fucosylation, low Fc galactosylation and low Fc sialylation based on their patent ^31^. In the meantime the company has performed clinical phase I ^32^ and II (NCT00952575; completed 2011) studies on roledumab ^33^. The phase II study was designed to demonstrate the ability of roledumab to effectively eliminate exogenously administered RhD-positive RBCs from the circulation of an RhD-negative individual, thereby preventing RhD allo-immunization. The results have not been published yet.

However, based on the findings described above regarding the effector functions of differentially glycosylated IgG Abs, it is questionable whether an anti-tumor IgG1 Ab and an anti-RhD IgG1 Ab should have the same Fc glycosylation. Whether low-galactosylated, low-sialylated RhD-specific IgG Abs can inhibit the induction of pathogenic immune reactions against RhD-positive fetal RBCs or rather enhance allo-immunization is also questionable. To identify the Fc galactosylation and sialylation of RhD-specific IgG Abs in a commercially available polyclonal anti-RhD IgG product, we purified RhD-specific IgG Abs from the approved product Rhophylac® (CSL Behring, King of Prussia, PA, USA) and analyzed the Abs’ Fc glycosylation.

## Methods

### Purification of RhD-specific IgG Abs from Rhophylac®

Total IgG from the commercial polyclonal anti-RhD IgG product Rhophylac® was purified using protein-G-sepharose (GE Healthcare, Fairfield, CT, USA). RhD-specific IgG Abs from the purified total IgG Abs of Rhophylac® were enriched using RhD-positive human erythrocytes. For this purpose, anonymous RhD-positive erythrocyte concentrates were obtained from the blood bank of the Charité - University Hospital Berlin. The erythrocyte concentrate was washed with 1 mM EDTA in PBS. Next, 20 ml of erythrocyte concentrate was diluted 1:1 with purified total IgG from Rhophylac® in PBS, which contained approximately 600 µg of RhD-specific IgG Abs as indicated by the company, and was incubated for 2h at 4°C. The erythrocytes were then washed five times with PBS. Subsequently, RhD-specific IgG Abs were eluted with 0.15 M glycine pH 3.0; neutralized with 1 M Tris/HCl pH 9.0 and dialyzed against PBS. Two independent RhD-specific IgG purifications (A and B) were done. Enrichment of the RhD-specific IgG Abs was verified by fluorescence-activated cell sorting (FACS) analysis. IgG Fc glycosylation was characterized through MALDI-TOF mass spectrometry (MS).

### FACS analysis

**Supplementary figure 1.**
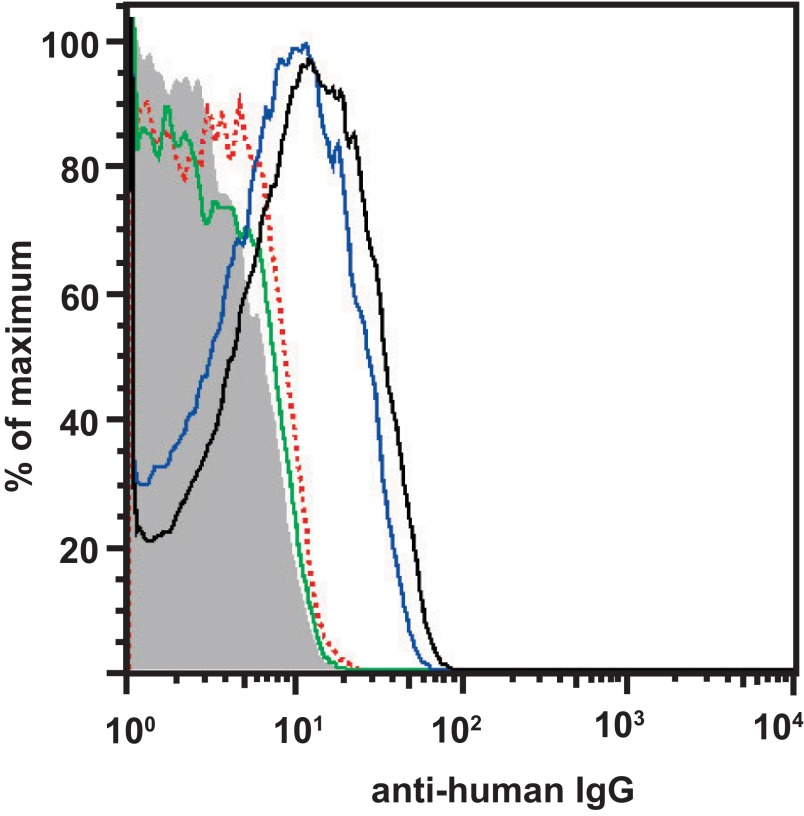
Verfication of the RhD-specific IgG purification. Purification of RhD-specific IgG Abs was verified by FACS analysis. RhD-positive human RBCs were stained with 260 µg/ml of total IgG from Rhophylac® (blue), 2 µg/ml purified RhD-specific IgGs (black; purification A) or 2 µg/ml of total IgG from Rhophylac® (red) and an anti-human IgG APC-coupled secondary Ab and analyzed by FACS analysis. The gray curve represents the unstained RBCs and the green curve represents the RBCs stained only with the secondary antibody. An overlay of different histograms, containing the analysis of the RhD-specific IgG Abs from purification A, is shown.

Enrichment of the purified polyclonal RhD-specific IgG Abs was verified by FACS analysis (FACS Calibur with CellQuest Pro software, version 6.0 (BD Biosciences, Franklin Lakes, NJ, USA)). RhD-positive human RBCs were stained with 260 μg/ml of total IgG from Rhophylac® (blue), 2 μg/ml purified RhD-specific IgGs (black) or 2 μg/ml of total IgG from Rhophylac® (red) and an anti-human IgG APC-coupled secondary Ab (#550931; BD Biosciences) in cold PBS containing 0.5% bovine serum albumin (Sigma-Aldrich; St. Louis, MO, USA). The stained cells were gated on the main erythrocyte population in a FSC/SSC blot to exclude fragments and aggregates and analyzed in an anti-human IgG (APC) histogram. The data were analyzed with FlowJo 7.2.5 from Tree Star, Inc. Ashland, Oregon, USA. An overlay of different histograms, containing the analysis of the RhD-specific IgG Abs from purification A, is shown in Supplementary figure 1. The FCS files can be found in the data files below.

FACS analyses of purified RhD-specific IgG antibodies unstained.001FACS analysis with Rhesus D (RhD)-positive erythrocytes to verify the enrichment of RhD-specific IgGs from total IgG of Rhophylac. The cells in this file are unstained. The cells were gated on the main erythrocyte population in a FSC/SSC blot to exclude fragments and aggregates, analyzed in an anti-human IgG (APC) histogram and shown in the overlay histogram in Supplementary Figure 1 in the main text.
only_secondary_antibody.004FACS analysis with RhD-positive erythrocytes to verify the enrichment of RhD-specific IgGs from total IgG of Rhophylac.The cells in this file are only stained with a secondary APC-coupled anti-human IgG antibody. The cells were gated on the main erythrocyte population in a FSC/SSC blot to exclude fragments and aggregates, analyzed in an anti-human IgG (APC) histogram and shown in the overlay histogram in Supplementary Figure 1 in the main text.
260_µg_per_ml_total_IgG_from_Rhophylac.002FACS analysis with RhD-positive erythrocytes to verify the enrichment of RhD-specific IgGs from total IgG of Rhophylac.The cells in this file are stained with 260 µg/ml total IgG from Rhophylac and subsequently with a secondary APC-coupled anti-human IgG antibody.The cells were gated on the main erythrocyte population in a FSC/SSC blot to exclude fragments and aggregates, analyzed in an anti-human IgG (APC) histogram and shown in the overlay histogram in Supplementary Figure 1 in the main text.
2µg_per_ml_purified_RhD_specific_IgG(from_purification_A).005FACS analysis with RhD-positive erythrocytes to verify the enrichment of RhD-specific IgGs from total IgG of Rhophylac.The cells in this file are stained with 2 µg/ml purified RhD-specific IgG from purification A and subsequently with a secondary APC-coupled anti-human IgG antibody. The cells were gated on the main erythrocyte population in a FSC/SSC blot to exclude fragments and aggregates, analyzed in an anti-human IgG (APC) histogram and shown in the overlay histogram in Supplementary Figure 1 in the main text.
2µg_per_ml_purified_RhD_specific_IgG(from_purification_B).007FACS analysis with RhD-positive erythrocytes to verify the enrichment of RhD-specific IgGs from total IgG of Rhophylac.The cells in this file are stained with 2 µg/ml purified RhD-specific IgG from purification B and subsequently with a secondary APC-coupled anti-human IgG antibody.These cells are not shown in the overlay histogram in Supplementary Figure 1 in the main text. However, the staining looks like the staining from the file before.
2_µg_per_ml_total_IgG_from_Rhophylac.003FACS analysis with RhD-positive erythrocytes to verify the enrichment of RhD-specific IgGs from total IgG of Rhophylac.The cells in this file are stained with 2 µg/ml total IgG from Rhophylac and subsequently with a secondary APC-coupled anti-human IgG antibody. The cells were gated on the main erythrocyte population in a FSC/SSC blot to exclude fragments and aggregates, analyzed in an anti-human IgG (APC) histogram and shown in the overlay histogram in Supplementary Figure 1 in the main text.

### Glycan analysis through MALDI-TOF MS

The purified total and RhD-specific IgG samples were hydrolyzed with recombinantly expressed endoglycosidase S (EndoS) from *Streptococcus pyogenes*, an enzyme that hydrolyzes N-glycans only from the Fc portion of the IgG Abs, to prevent the analysis of glycans from pontential contaminating RBC proteins ^34^. The resulting N-glycans were purified through solid-phase extraction using reversed-phase C18 and graphitized carbon columns (Alltech, Deerfield, IL, USA), permethylated and further investigated by MALDI-TOF MS ^21^. The spectra were recorded on an Ultraflex III mass spectrometer (Bruker Corporation, Billerica, MA, USA) equipped with a Smartbeam laser. Calibration was performed on a glucose ladder, and 2,5-dihydroxybenzoic acid (DHB) was used as the matrix. Spectra were recorded in reflector positive ionization mode and mass spectra from 3,000 laser shots were accumulated. The crude glycan analysis data of the two independent RhD-specific IgG Ab purifications A and B can be found in the data files below.

**Supplementary figure 2.**
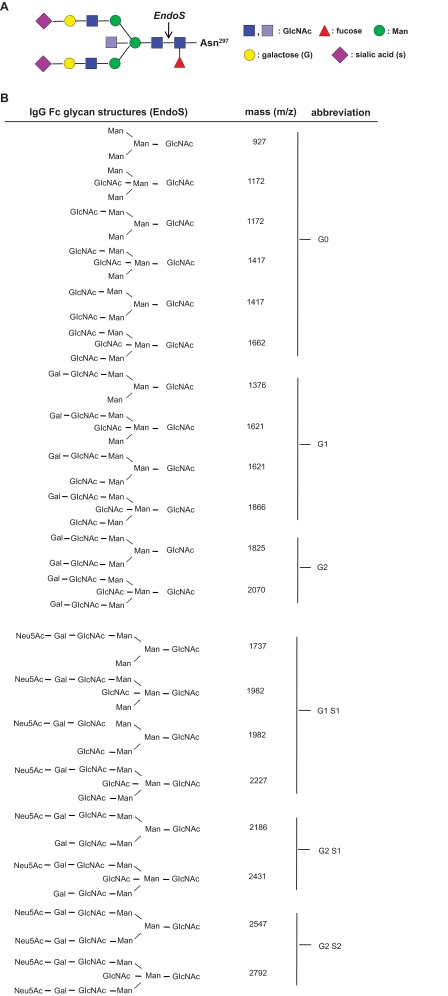
EndoS-released glycan structures. ( **A**) The biantennary IgG Fc glycan core structure, which is coupled to Asn 297 consists of two N-acetyl-glucosamines (GlcNAc; dark blue) and three mannoses (Man), which can be further decorated with fucose; bisecting GlcNAc (light blue) and terminal GlcNAc (dark blue), galactose (G, Gal) and sialic acid (N-acetylneuraminic acid (Neu5Ac), S, Sial). The cleavage site of EndoS is indicated by an arrow. ( **B**) The molecular mass (m/z) of the possible Fc glycan structures (permethylated) released from Asn 297 upon EndoS treatment.

MALDI-TOF MS glycan analysis of RhD-specific IgG antibodies (crude data) Series_A_09037-013_total_IgG.csvMALDI-TOF MS glycan analysis of total IgG from Rhophylac, which was the starting material for the purification of Rhesus D (RhD)-specific IgG antibodies (purification A). The area under the peak is shown for each selected molecular mass (m/z) and presented as percentage of all the indicated area values. The glycan structure and abbreviation for each m/z is shown in Supplementary Figure 2 and the summary of all MALDI-TOF MS analyses is shown in Figure 1 in the main text.
Series_A_09037-012_RhD-specific_IgG.csvMALDI-TOF MS glycan analysis of RhD-specific IgG antibodies (purification A). The area under the peak is shown for each selected molecular mass (m/z) and presented as percentage of all the indicated area values. The glycan structure and abbreviation for each m/z is shown in Supplementary Figure 2 and the summary of all MALDI-TOF MS analyses is shown in Figure 1 in the main text.
SeriesB_09037-192a total_IgG.csvMALDI-TOF MS glycan analysis (measurement a of a duplicate measurement) of total IgG from Rhophylac, which was the starting material for the purification of RhD-specific IgG antibodies (purification B). The area under the peak is shown for each selected molecular mass (m/z) and presented as percentage of all the indicated area values. The glycan structure and abbreviation for each m/z is shown in Supplementary Figure 2 and the summary of all MALDI-TOF MS analyses is shown in Figure 1 in the main text.
SeriesB_09037-192b total_IgG.csvMALDI-TOF MS glycan analysis (measurement b of a duplicate measurement) of total IgG from Rhophylac, which was the starting material for the purification of RhD-specific IgG antibodies (purification B). The area under the peak is shown for each selected molecular mass (m/z) and presented as percentage of all the indicated area values. The glycan structure and abbreviation for each m/z is shown in Supplementary Figure 2 and the summary of all MALDI-TOF MS analyses is shown in Figure 1 in the main text.
Series_B_09037-191a_RhD-specific_IgG.csvMALDI-TOF MS glycan analysis (measurement a of a duplicate measurement) of RhD-specific IgG antibodies (purification B). The area under the peak is shown for each selected molecular mass (m/z) and presented as percentage of all the indicated area values. The glycan structure and abbreviation for each m/z is shown in Supplementary Figure 2 and the summary of all MALDI-TOF MS analyses is shown in Figure 1 in the main text.
SeriesB_09037-191b RhD-specific_IgG .csvMALDI-TOF MS glycan analysis (measurement b of a duplicate measurement) of RhD-specific IgG antibodies (purification B). The area under the peak is shown for each selected molecular mass (m/z) and presented as percentage of all the indicated area values. The glycan structure and abbreviation for each m/z is shown in Supplementary Figure 2 and the summary of all MALDI-TOF MS analyses is shown in Figure 1 in the main text.

## Results and discussion

To assess the Fc galactosylation and sialylation of RhD-specific IgG Abs in a commercially available polyclonal anti-RhD IgG product, we purified RhD-specific IgG Abs from Rhophylac® and analyzed the Abs’ Fc glycosylation ( Figure 1, Supplementary figure 1 and Supplementary figure 2 and the data files). We found that the purified polyclonal RhD-specific IgG Abs were even more galactosylated and sialylated than the total Rhophylac® IgG Abs ( Figure 1, Supplementary figure 1 and Supplementary figure 2 and the data files), which are comparable to total IgG Abs in immunosuppressive IVIG. The injection of RhD-positive RBCs into RhD-negative men obviously induced no pathogenic immune response. Comparable results have recently been generated by analyzing allergen-specific human IgG Abs after successful allergen-specific immunotherapy for birch pollen allergy ^21^. Together, these results further indicate that tolerance induction induces mainly galactosylated and sialylated IgG Abs that have the potential to inhibit pathogenic immune responses via ICs ^21– 24^.

These results strongly suggest that only galactosylated and sialylated, immunosuppressive monoclonal RhD-specific IgG Abs can be substituted for polyclonal anti-RhD IgG products to inhibit pathogenic allo-immunity in RhD-negative pregnant woman. Low fucosylated, low agalactosylated and low sialylated RhD-specific IgG Abs might not only enhance pro-inflammatory immune responses against RhD-positive fetal RBCs but also have the potential to attack RhD-positive fetal RBCs after transplacental passage.
