# Supplementary material for: Anti-rhesus D prophylaxis in pregnant women is based on sialylated IgG antibodies
Source: F1000Res. 2013 Aug 9;2:169. [Version 1] doi: 10.12688/f1000research.2-169.v1 (PMC3829133; doi:10.12688/f1000research.2-169.v1)
Supplement: Peer review history

# Peer review: Referee response for version 1

**Published:** 2013-09-27

**Authors:** Mikael Karlsson

**Affiliations:** 1 Department of Medicine-Solna, Karolinska Institutet, Karolinska University Hospital Solna, Stockholm, Sweden

Anti-RhD therapy is one of the most successful antibody therapies ever invented and still we do not know how antibodies block Rh immunization. Recently, a novel level of regulation through alterations in glycosylation patterns has been started to be appreciated. This study is a good starting point to investigate if this mechanism of regulation by antibodies is involved. The glycosylation pattern found in the anti-RhD pool of antibodies suggests that an active tolerogenic pathway is involved and encourages further investigation.

I have read this submission. I believe that I have an appropriate level of expertise to confirm that it is of an acceptable scientific standard.

# Peer review: Referee response for version 1

**Published:** 2013-09-23

**Authors:** Tracy McGaha

**Affiliations:** 1 Cancer Immunology, Inflammation, and Tolerance Program, Georgia Regents University Cancer Center, Augusta, GA, USA

In the article “Anti-rhesus D prophylaxis…” by Winkler, Berger, and Ehlers the authors present data demonstrating enrichment of galactosylation and sialylation in the anti Rh-D fraction of Rhophylac. This study is well written with adequate discussion of methods. Moreover, the report builds on exciting recent findings that the pattern of glycosylation is an important determinant factor in the effector function of IgG providing mechanistic control of FcGR interactions and tolerogenic v.s. inflammatory immunity.Their results would suggest the therapeutic value of anti-RhD therapy may lie in an active tolerogenic response rather than via clearance mechanisms, a theory which should be tested moving forward.

The title is a bit misleading as it suggests experimental evidence contained within the report demonstrates anti-RhD activity is due to glycosylation, a theorized outcome that has yet to be demonstrated. A revision to reflect this fact may be appropriate.

Otherwise, it is an exciting report pointing to a novel mechanism that may drive prophylactic anti-RhD therapy.

I have read this submission. I believe that I have an appropriate level of expertise to confirm that it is of an acceptable scientific standard.

# Response

**Published:** 2013-10-23

**Authors:** André Winkler

**Affiliations:** University of Luebeck, Germany

Dear Tracy,

many thanks for reviewing our manuscript.

I agree that the title is a bit misleading. I would propose to slightly change it to: " *Anti-rhesus D prophylaxis in pregnant women is might based on sialylated IgG antibodies*"

This statement is based on the fact that AntiD IgG are highly sialylated and the immunoregulatory properties of sialylated IgG has been shown previously by us and by others.

Do you agree?

# Response

**Published:** 2013-10-28

**Authors:** Tracy McGaha

**Affiliations:** Georgia Regents University, USA

Dear Dr Winkler,

I think the proposed revision is appropriate and acceptable.

Regards,

Tracy McGaha.
